# Pokémon Go and Exposure to Mosquito-Borne Diseases: How Not to Catch ‘Em All

**DOI:** 10.1371/currents.outbreaks.2d885b05c7e06a9f72e4656d56b043cd

**Published:** 2016-11-15

**Authors:** Rachel J. Oidtman, Rebecca C. Christofferson, Quirine A. ten Bosch, Guido Espana, Moritz U. G. Kraemer, Andrew Tatem, Christopher M. Barker, T. Alex Perkins

**Affiliations:** Department of Biological Sciences and Eck Institute for Global Health, University of Notre Dame, Notre Dame, Indiana, USA; Department of Pathobiological Sciences, School of Veterinary Medicine, Louisiana State University, Baton Rouge, Louisiana, USA; Department of Biological Sciences and Eck Institute for Global Health, University of Notre Dame, Notre Dame, Indiana, USA; Department of Biological Sciences and Eck Institute for Global Health, University of Notre Dame, Notre Dame, Indiana, USA; Department of Zoology, University of Oxford, Oxford, United Kingdom; WorldPop, Department of Geography and Environment, University of Southampton, Southampton, United Kingdom; Flowminder Foundation, Stockholm, Sweden; Department of Pathology, Microbiology, and Immunology, School of Veterinary Medicine, University of California, Davis, California, USA; Department of Biological Sciences and Eck Institute for Global Health, University of Notre Dame, Notre Dame, Indiana, USA

## Abstract

*Pokémon Go* is a new game that encourages players to venture outdoors and interact with others in the pursuit of virtual *Pokémon* characters. With more time spent outdoors overall and in sometimes large congregations, *Pokémon Go* players could inadvertently elevate their risk of exposure to mosquito-borne diseases when playing in certain areas at certain times of year. Here, we make an initial assessment of the possible scope of this concern in the continental United States, which experiences its highest seasonal transmission of West Nile, Zika, and other viruses during summer and early fall. In particular, we propose that the times of day when many disease-relevant mosquito species are most likely to engage in blood feeding coincide with times of day when *Pokémon Go* activity is likely to be high, and we note that locations serving as hubs of *Pokémon Go* activity may in some cases overlap with areas where these mosquitoes are actively engaged in blood feeding. Although the risk of mosquito-borne diseases in the continental U.S. is low overall and is unlikely to be impacted significantly by *Pokémon Go*, it is nonetheless important for *Pokémon Go* players and others who spend time outdoors engaging in activities such as barbecues and gardening to be aware of these ongoing risks and to take appropriate preventative measures in light of the potential for outdoor activity to modify individual-level risk of exposure. As *Pokémon Go* and other augmented reality games become available in other parts of the world, similar risks should be assessed in a manner that is consistent with the local epidemiology of mosquito-borne diseases in those areas.

## The *Pokémon Go* craze and its implications for public health

*Pokémon Go* is a game premised on interactions between the real world and a virtual *Pokémon* world via an application on the user’s mobile phone. The objective of the game is to catch 151 different *Pokémon* characters, which can be found at various publicly accessible real-world locations, such as parks, businesses, bodies of water, and numerous other locations in between^1^. As of July 11, 2016, 5.9% of all Android users in the United States, or about 6.4 million people, had not only downloaded *Pokémon Go* but played it daily since its release on July 6, 2016^2^^,^ 3. Since the end of August, the number of *Pokémon Go* daily users has diminished as interest in the game has waned, daylight hours have reduced, and weather conditions have become less conducive to spending time outdoors^4^. Despite waning interest, one clear outcome of the *Pokémon Go* phenomenon in summer 2016 was that millions of people spent more time outdoors - traveling farther and wider to “catch ‘em all” - than they might have otherwise. Another is that there were large groups congregating together at “hot spots” of relevance to the game, creating anomalous concentrations of people that ordinarily would not occur. Although the initial *Pokémon Go* fad may have reached its conclusion, it and other games in the new augmented reality genre could induce similar changes in human behavior in future summers.

There are many benefits of spending more time outdoors, including higher levels of physical activity with associated positive health outcomes such as reduced rates of obesity and depression^5^^,^ 6 ^,^ 7.* Pokémon Go* players undoubtedly enjoy many benefits of playing this game and spending more time outdoors, but at the same time there have been numerous reports of risks to personal health and safety associated with playing *Pokémon Go*, including armed robbery, traffic collisions, and various other accidents as extreme as walking off a 90-foot cliff^8^^,^ 9 ^,^ 10. Here, we draw attention to another possible risk of playing *Pokémon Go*: increased exposure to mosquito bites and to the pathogens that they transmit in certain areas and at certain times of year. Although *Pokémon Go* has yet to be released in some countries with the greatest risk of mosquito-borne pathogens, the timing of its release occurred just as the continental U.S. was entering a seasonally high period of increased risk for transmission and/or introduction of mosquito-borne pathogens^11^^,^ 12 ^,^ 13.

**The when, where, and who of mosquito-borne pathogen transmission**

A number of mosquito-borne pathogens, especially viruses, pose a recurring seasonal risk to individuals who spend more time outdoors in summer months in the continental U.S. These include West Nile virus (WNV), Eastern equine encephalitis virus (EEEV), St. Louis encephalitis virus (SLEV), and La Crosse virus (LACV), all of which are maintained in enzootic cycles involving multiple non-human vertebrate and mosquito species. Because humans are dead-end hosts for transmission of these viruses^14^^,^ 15 ^,^ 16, transmission to humans ultimately results from infections among non-human vertebrates on which various mosquito species regularly engage in blood-feeding^11^. Outdoor activity near key habitats for those species may elevate the risk of exposure to these viruses^17^^,^ 18 ^,^ 19 ^,^ 20.

The timing of outdoor activity is also important, as different vector species tend to bite at different times of day, with the vector of LACV biting during the day^21^, vectors of WNV and SLEV generally biting in the hours around and after dusk^22^^,^ 23 ^,^ 24, and the multiple vectors of EEEV biting at a variety of times. Outdoor activity by humans at these times has been documented as a risk factor for both WNV^25^ and LACV^20^ infections. Tweets from mobile devices using the case-insensitive string “#pokemongo” also appear to occur most frequently in the hours before and around dusk (Fig. 1). Although some portion of these tweets are likely composed indoors, it is also likely that many of these tweets are composed outdoors as players interact with each other. Much like mobile phone records and other digital data, twitter activity can serve as a proxy of time allocation in different areas^26^. To the extent that tweets from mobile devices in Fig. 1 serve as a proxy for time spent outdoors, it appears that there may be elevated *Pokémon Go* activity around the time that many people take lunch breaks and in the hours after work but before dusk. Some outdoor activity may continue around dusk and shortly after, but then drop to its lowest levels during late night and early morning hours (Fig. 1).

**Timing of mosquito biting and #pokemongo tweets:**
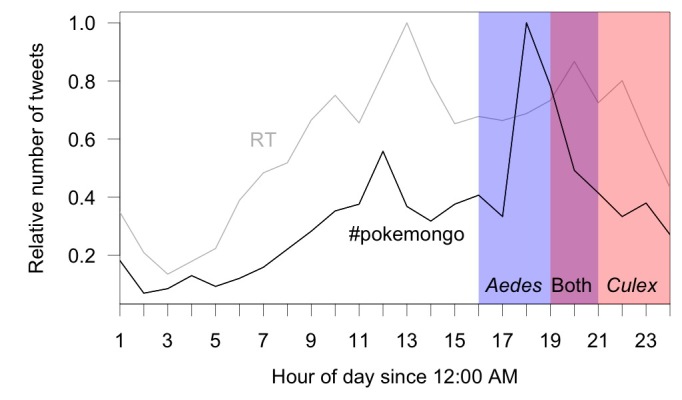
Number of tweets from mobile devices by hour relative to the daily maximum in Miami, Florida (summed over July 17-24, 2016) with the gray line representing tweets mentioning “RT” (a commonly used string indicating a re-tweet) and the black line representing tweets mentioning "#pokemongo.” The shaded areas provide a crude indication of heightened times of biting activity for *Ae. albopictus* and *aegypti* (blue), *Cx. tarsalis*, *quinquefasciatus*, and *pipiens* (red), or both (purple)^23^^,^ 24 ^,^ 27 ^,^ 28 ^,^ 29. Tweets were downloaded using the Perl library Net::Twitter^30^, and all code that was used to make this figure is https://github.com/confunguido/PokemonDataAnalysis.

With the potential for increased contact between humans and certain mosquito vectors also comes an increased risk of sporadic transmission of non-endemic tropical diseases in the continental U.S., including dengue (DENV), chikungunya (CHIKV), and Zika (ZIKV) viruses. Within the last several years, there have been short chains of local transmission of DENV, CHIKV, and most recently ZIKV in the continental U.S., with autochthonous, mosquito-borne transmission of ZIKV documented recently in several neighborhoods in Miami, Florida^31^^,^ 32 ^,^ 33. Mathematical models suggest that a key factor in the size of these transmission chains and the risk to individuals is the rate of contact between people and mosquitoes^34^^,^ 35. *Aedes* species (*Ae. aegypti* and *Ae. albopictus*) transmit these viruses^12^^,^ 36 and bite during a broad range of times throughout the day, but especially in the hours before dusk^28^^,^ 29. Given the relatively catholic blood-feeding preferences of *Ae. albopictus*, exposure to biting by these mosquitoes depends on the relative abundance of non-human vertebrate host species within an area^29^^,^ 37 ^,^ 38.

*Pokémon Go* activity is often concentrated consistently over time around specific locations known as *PokéStops* and *Pokémon* Gyms^1^ (Fig. 2). *PokéStops* are locations that players visit to collect necessary items that aid in collecting more *Pokémon*, and are generally located in public outdoor spaces, such as art installations, monuments, and parks^1^ 39 ****. *Pokémon* Gyms are outdoor locations that players visit to train their *Pokémon* and to interactively battle one another, spending prolonged periods of time outside around other players^40^ ****. The extent to which *PokéStops* and *Pokémon* Gyms overlap with areas where *Ae. aegypti* and *Ae. albopictus* are actively engaged in blood feeding is likely to vary considerably, with locations near parks or residential areas generally having the highest possibility of overlap. In such locations, one relevant possibility is that human congregation around these locations could allow for a shift towards more frequent biting on humans and potentially longer transmission chains of pathogens transmitted by these mosquitoes.

**Outdoor congregation of Pokémon Go players:**
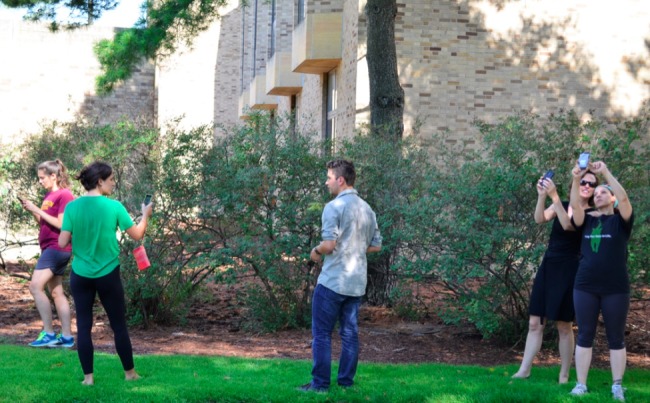
A group of *Pokémon Go* players congregate to catch *Pokémon*. Congregation such as this is an inevitable aspect of playing the game. *Pokémon* are more likely to appear near *PokeStops*, and players frequent *Pokémon* Gyms to engage in interactive play with others^1^. (Photo credit: Mauna Dasari)

Given recent transmission of ZIKV in the Miami area, we examined the extent of spatial overlap between *PokéStops* and *Pokémon* Gyms (obtained from https://mapokemon.com) and portions of the city where mosquito-borne transmission of ZIKV has been documented (Fig. 3). We found at least two such locations at the edges of one of these neighborhoods, several more within one mile, and dozens more within the Miami area as a whole. Overlaying a map of *Ae. aegypti* occurrence probabilities onto these locations, we found that the probability that *Ae. aegypti* occur in the general vicinity of these areas ranges 0.92-0.96^36^. Although this analysis provides no evidence of a role of *Pokémon Go* activity in the Zika outbreak in Miami, it does highlight the potential for spatial overlap between areas of *Pokémon Go* activity and mosquito-borne virus transmission. Furthermore, because the occurrence probability maps that we used are intended primarily for comparative purposes at broad geographic scales rather than for associative studies at the finer scales at which *Pokémon Go* activity takes place, the juxtaposition of these occurrence probabilities with *PokéStops* and *Pokémon* Gyms serves only as a general reminder of the presence of *Ae. aegypti* throughout the area. Just like anyone else spending time outdoors in these areas, individuals playing *Pokémon Go* should heed cautions from the Centers for Disease Control and Prevention for reducing exposure to ZIKV and other viruses in the context of an active outbreak. At the same time, it is worth bearing in mind that risk of exposure to ZIKV or other viruses around the vast majority of *PokéStops* and *Pokémon* Gyms will usually be negligible. In those cases, the risk of exposure to one of these viruses should generally not outweigh the health benefits of increased time outdoors that is associated with playing *Pokémon Go* 41 ^,^ 42.

**PokéStops and Pokémon Gyms in Miami:**
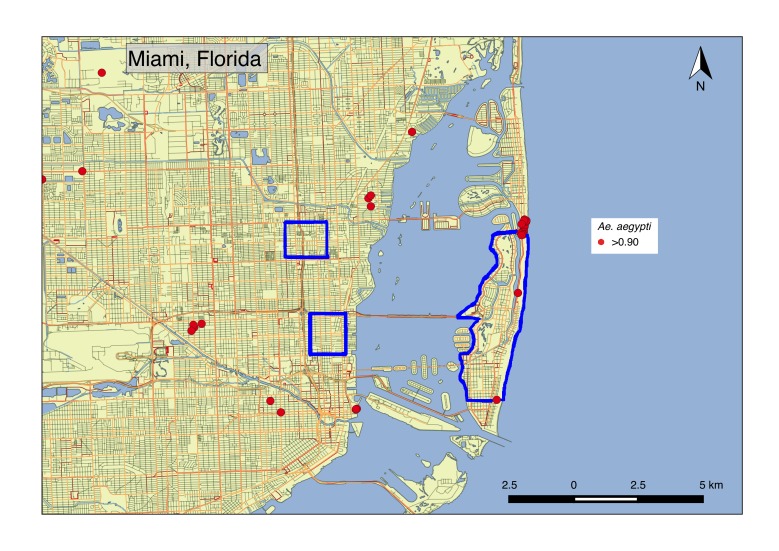
Google Maps satellite image of Miami, Florida. The area of each red dot shows the probability of occurrence of *Ae. aegypti* within the 5 km x 5 km grid cell in which a given *PokéStop* or *Pokémon* Gym has been geo-located by https://mapokemon.com. These probabilities do not necessarily correspond to probabilities that *Ae. aegypti* occur specifically at *PokéStops* and *Pokémon* Gyms, or that they engage in contact with individuals playing *Pokémon Go*. Instead, they serve as a reminder of the uniformly high probability across the Miami area that* Ae. aegypti* are generally present. The regions outlined in blue demarcate neighborhoods in Miami-Dade County, FL that had experienced local ZIKV transmission as of October 31, 2016^33^. Map was created with RgoogleMaps^43^. *PokéStop* and *Pokémon* Gym location data provided by Alexander Wigmore from https://mapokemon.com.

Even if spending more time outdoors playing *Pokémon Go* could result in higher exposure to mosquito-borne viruses, this may not necessarily result in higher incidence of overt disease associated with mosquito-borne virus infection. Many infections of humans by these viruses are asymptomatic, and those that are not tend to occur with higher probability in particular age groups. Specifically, LACV infection poses a greater risk of neuroinvasive disease to children, and WNV infection poses a greater risk of neuroinvasive disease to older adults^44^. Approximately 9 out of 10 *Pokémon Go* players are below age 55 and nearly 7 out of 10 are below age 35^45^, suggesting that *Pokémon Go* players who become infected with WNV may generally be at relatively low risk of experiencing neuroinvasive disease. SLEV and EEEV infections pose a more even risk of neuroinvasive disease with respect to age, although their absolute risk is very low^44^. For DENV, CHIKV, and ZIKV, the risk of disease by age depends on the history of transmission and the extent of immunity within a population. Because there is virtually no immunity to these viruses in the U.S., any autochthonous transmission that happens could result in symptomatic disease in individuals of any age. Direct associations between playing *Pokémon Go* and acquiring these diseases have not yet been formally investigated, however.

**Having fun, staying safe, and leveraging novel data streams for public health**

We have drawn attention to persistent and emerging mosquito-borne disease risks in the continental U.S. and have noted their association with time spent outdoors^25^^,^ 46. Although increased time outdoors playing *Pokémon Go* could elevate the risk of acquiring these diseases, it is important to note that the risk of acquiring these diseases within the continental U.S. is extremely low in an absolute sense and that *Pokémon Go* activity is not expected to ever become a major driver of transmission relative to other factors^31^^,^ 44. Nonetheless, it is important for *Pokémon Go* enthusiasts and others who spend time outdoors in areas and at times of heightened mosquito biting to be aware of these risks and to take proper precautions per Centers for Disease Control and Prevention guidelines^47^, including wearing long sleeves and pants when possible and applying insect repellents with DEET or another recommended active ingredient. Surveys conducted in the continental U.S. suggest that there is a need for improved education around these issues^48^.

In addition to personal protection, there may be actions that the developers of *Pokémon Go* and similar games could consider in partnership with health authorities to help mitigate these risks. For example, in-app alerts could be used to notify players of local, seasonally relevant mosquito-borne disease risks, or to remind players to apply insect repellent. In some cases, it may also be advisable for *Pokémon* Gyms or *PokéStops* to be relocated if a specific risk emerges, such as the ZIKV outbreak in Miami or in the event mosquitoes test positive for a virus such as WNV. Although *Pokémon Go* activity has peaked for now^49^, these considerations remain relevant for those who will play this and future offerings in the augmented reality space in coming summers.

As *Pokémon Go* encourages people to spend more time outdoors actively using their mobile phones, at times in large groups, unique research opportunities may also emerge. A wide variety of novel data streams are increasingly applied to address public health challenges^50^^,^ 51, such as the use of Twitter data to model influenza dynamics^52^. Similarly, geo-referenced, time-stamped data about *Pokémon Go* activity could provide a rich data source on spatial and temporal patterns of indoor and outdoor time use. To the extent that associations might exist between congregations of *Pokémon Go* players and mosquito-borne disease risk, future work could address the extent to which other outdoor congregations at times of high mosquito biting activity, such as outdoor movies and barbecues, might pose an elevated risk of exposure to mosquito-borne diseases. In conclusion, as millions of people continue to explore augmented reality games such as *Pokémon Go* each day, it will be important for public health officials and others to think creatively about how to minimize risks and maximize opportunities associated with this fundamentally new and distinct activity.

## Competing Interest Statement

The authors have declared that no competing interests exist.

## Ethics Statement

The individuals in this manuscript have given written informed consent (as outlined in PLOS consent form) to publish these case details.

## Corresponding Author

Rachel Oidtman, E-mail: roidtman@nd.edu

Alex Perkins, E-mail: taperkins@nd.edu

## Data Availability Statement

Data provided by: https://mapokemon.com from Alexander Wigmore
